# Learning from Sisyphus: time to rethink our current, ineffective strategy on neurodevelopmental environmental toxicants

**DOI:** 10.1186/s12940-020-00587-w

**Published:** 2020-03-11

**Authors:** Larissa Takser, Darel John Hunting

**Affiliations:** 1 Département de Pédiatrie, Faculté de Médecine et Sciences de la Santé, Université de Sherbrooke, 3001, 12e avenue Nord, Sherbrooke (Qc), J1H 5N4 Canada; 2 Département de Psychiatrie, Faculté de Médecine et Sciences de la Santé, Université de Sherbrooke, Sherbrooke (Qc), Canada; 3 Département de Médecine Nucléaire et Radiobiologie, Faculté de Médecine et Sciences de la Santé, Université de Sherbrooke, 3001, 12e avenue Nord, Sherbrooke (Qc), J1H 5N4 Canada

**Keywords:** Neurotoxicity, Environmental epidemiology, Knowledge transfer, Neurodevelopmental disorders, Low dose exposures

## Abstract

**Background:**

The overwhelming number of potentially toxic chemicals in consumer products and in our daily environment makes it unrealistic to carry out in-depth analyses of each product with the objective of banning and eliminating toxic chemicals from our environment.

**Objectives:**

To present the challenges that environmental toxicology and epidemiology are currently facing in the context of ubiquitous chemical pollution.

**Discussion:**

We propose a realistic and pragmatic approach to this Herculean problem.

## Background

Research in Toxicology and Environmental Epidemiology is an important contributor to primary prevention and public health by identifying and characterizing environmental hazards. There is an ongoing debate on the question of whether or not Toxicology delivers the evidence in a sufficiently efficient and timely manner for decision making in public health. One of the issues identified is the need to adopt evidence-based principles and methods. Historically, all toxicological research is almost obsessively focused on decision making in regards to controlling or banning chemicals. This has resulted in some spectacular and long lasting successes, including the banning of lead in gasoline and controls on mercury exposure. However, the realities we are currently addressing are qualitatively different from those which were addressed by classic toxicology a few decades ago.

In a reality of thousands of new chemicals and limited resources, we are spending millions to generate knowledge on the toxicity of many environmental chemicals in the hope of banning a few selected chemicals. Low dose exposures to mixtures of modern chemicals is a major challenge in epidemiology/toxicology and necessitates a new approach. The banning of bisphenol A (BPA), which has estrogenic activity, from certain consumer products such as baby bottles took years of research and lobbying. But now, do we restart the process with bisphenol B (BPB), bisphenol S (BPS), bisphenol F (BPF) etc.?

It is this approach the best investment of our time and money and does it offer the best protection of our fellow citizens and the environment?

In 2007, the European Union put in place the REACH regulation (Registration, Evaluation, Authorization and Restriction of Chemicals). The aim is to protect health and the environment. Of interest to consumers, is the requirement that retailers provide, upon request, information about the chemicals present in their products. The REACH regulation is extremely complex and ambitious and would use very large numbers of animals for toxicology tests [1]. However, the procedures are unlikely to identify neurodevelopmental toxicants or the effects of chronic exposure to mixtures of toxicants.

## How much knowledge do we need to protect us from an environmental hazard?

Modern toxicology and environmental health science have been built on a largely accepted premise that scientific knowledge on the toxic effects of environmental contaminants and risk assessment will improve our quality of life through preventive legislative actions, such as controlling (e.g. guidelines for arsenic in drinking water) or banning chemicals. This premise presupposes that all environmental risks are manageable and that the knowledge we generate will eventually be translated into action. We cite the example of the discovery of the neurotoxic effects of lead on generations of young children, followed by the successful banning of the use of lead in gasoline and residential paint, as a historical victory of environmental epidemiology and public health. However, the recent stories of lead in drinking water (Flint, MI, and Newark, New Jersey, USA) [2, 3] or non-banning of chlorpyrifos [4], call into question the limits of the application of existing knowledge as well as our role as scientists in the protection of our communities. How is it possible that we still have entire communities contaminated with “old” chemicals such as lead, which costs less than $1.00 to quantitate in blood and for which we know virtually everything about the sources, mechanism of action and dose-response curve? Why in tertiary community hospitals, do we not routinely consider levels of lead in the blood as a possible cause of neurodevelopmental disorders, when it should be at the top of the list?

Considering the overwhelming amount of knowledge on lead, the current examples of lead poisoning demonstrate that the amount of existing knowledge does not always correlate with the application of this knowledge by political decision makers, public health authorities, and medical care providers. Lead and mercury are examples of scientific overkill, where we continue to study these toxicants and use cases of public health failures, such as Flint (lead) and Grassy Narrows (mercury [5]) as justifications for more studies. Admittedly, some additional studies on exposure pathways and prevention may be justified. As scientists who aim to generate knowledge on low-dose exposures to “new toxins” we realize that there are serious obstacles to the production of valid knowledge for widespread low-dose environmental contaminants, which have effects far more subtle than lead and mercury. Modern toxicologists and epidemiologists need to be sure that the knowledge we generate today with public money will be translated into an action within a reasonable timeline. Training students in Environmental Toxicology to suffer the same fate as Sisyphus is not an attractive perspective. Current trainees in Toxicology will almost certainly address issues we cannot yet envision; however, we think they need to be exposed to the new paradigm of low, chronic exposures to multiple toxicants.

## Current versus classical environmental epidemiology: methodological and conceptual challenges

### No more “smoking gun”

Historically, Environmental toxicology and epidemiology have dealt with situations involving exposures to toxicants and specific symptoms. Classical examples are Minamata disease, caused by mercury, and mental retardation due to lead poisoning. The Holy Grail of classic epidemiology was the identification of the “smoking gun” and elimination of toxic substances from our environment. Decades later, when dealing with “new”, ubiquitously present chemicals, it is now clear that chasing toxic contaminants is like the game of Whack-A-Mole: after many years of research and lobbying, governments ban or restrict one substance, which is immediately replaced by several new ones with unknown toxic properties (ex: replacement of brominated flame retardants PBDEs (polybrominated diphenyl ethers) by emerging ones). Also, as pointed out by Grandjean and Landrigan in 2006 [6], we have progressively moved from studying poisoning incidents to studying subclinical effects in the entire population.

For many chemicals presently under investigation by academic researchers (i.e. phthalates, flame retardants), by the time we began studying them they were already ubiquitously present in human samples. Experimental animals in institutional facilities are also ubiquitously exposed to these chemicals [7]. Both situations mean that we must study the toxic effects of these chemicals without true negative control groups.

### Focus on neurodevelopmental phenotypes

In addition to studying ubiquitously exposed populations, which precludes the inclusion of unexposed controls, we are studying complex neurodevelopmental disorders (NDD), such as Attention Deficit/Hyperactivity Disorder (ADHD), autism, learning deficit etc. Conceptually, we are not investigating the toxicity of a specific substance, but rather a complex clinical entity with multifactorial etiology. Studying subtle effects on brain function is far more complex than for other organs and the phenotypes are less well characterised than for other biological systems, with the possible exception of the immune system.

In terms of methodology, we are moving from neurological phenotypes (e.g. Minamata disease) toward behavioral phenotypes, which implies different outcome measures (i.e. measures of motricity, attention or memory versus diagnostics and questionnaires based on the criteria of the Diagnostic and Statistical Manual of Mental Disorders (DSM-5) or designed to evaluate functional capacity). From a clinical point of view, these neurobehavioral phenotypes are very complex not only to study and to interpret in the context of a research project, but also to diagnose for the following reasons:

1. there is huge overlap between clinical entities, diagnoses, and disorders, which means that it is very difficult to identify a child as uniquely an ADHD because of the significant presence of co-morbidities. However, frequently, a child with co-morbidities will have one main diagnosis;
2. there are no objective biological tests which could help to discriminate clinical entities; diagnoses are based on behavioral reports by parents and, sometimes, on direct observation by clinicians (called “clinical diagnosis”). The fact that DSM-5 entities are not based on specific brain functions with known pathophysiological mechanisms was recently addressed by theNational Institutes of Health (NIH) [8];
3. diagnosis of relatively mild NDD, such as non severe ADHD cases, is highly influenced by social context which determines if a child functions or not in a given environment (i.e. strict academic environment versus home schooling); and
4. NDD probably result from a combination of several causal factors, which are still poorly understood (i.e. genetic, childhood abuse, infections, epigenetic, nutrition, microbiome, environmental contaminants, exposure to screens), which implies that we introduce a confounding bias due to unmeasured or unknown confounders.

Current environmental epidemiology is undergoing a deep qualitative transformation. We are correlating ubiquitous exposures with highly prevalent and not well defined phenotypes. Therefore, current environmental epidemiology has little chance of discovering a “smoking gun” and deals with low dose exposures. In addition, classical concepts like NOAEL (No Observable Adverse Effect Level) and negative (unexposed) controls are difficult to apply to poorly defined phenotypes and ubiquitous exposures. From a methodological perspective, we are more and more precise in exposure assessment (high performance analytical tools) and less and less precise in the assessment of effects or outcomes (behavioral questionnaires with few response options: “never/sometimes/often/almost all the time”). Finally, the neurodevelopmental effects of a given product may be incorrectly attributed to the active ingredient rather than to a contaminating toxicant (e.g. herbicide 2,4-dichlorophenoxyacetic acid contaminated by dioxins).

### Barely reproducible results

#### Type III error

The new reality of toxicology has dramatic repercussions on the lack of coherence and non-reproducibility of results from human studies. This non-reproducibility, which results from Type III errors,^1^ low precision epidemiological tools, and low external validity, decreases the credibility of new studies and nourishes the doubt and controversy about potential toxic effects with subsequent delays in decision making and action.

**Fig. 1:**
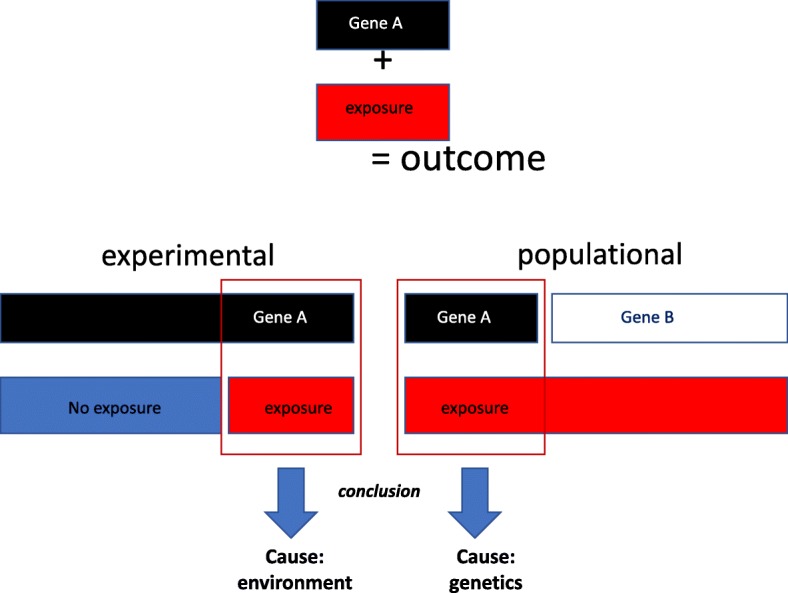
In animal experiments (experimental), both the genetic background and exposure to environmental toxicants can be controlled which allows one to test the toxic effects of specific contaminants. In human population (populational), virtually everyone may be exposed and thus “bad genes” may be blamed, since only carrier of gene A will be susceptible to the contaminant

Ubiquitous exposures are not only challenging from an analytical perspective, but can also create the illusion that another cause is related to the index outcome in cases of multifactorial causality. This phenomenon is called Type III error [9]. In the context of environmental toxicology, this phenomenon could explain why results on toxicity from experimental studies are barely reproducible in human populations (Fig. 1). In experimental studies, we deal with inbred animals and testing different exposure levels can reveal toxic effects. In contrast, in an ubiquitously exposed human population, with a range of genotypes, we may inconsistently conclude, based on dichotomic reasoning, that genetics is the causal factor rather than the environment. In reality, the right interpretation would be that the combination of both is harmful and that people with specific genetics should be protected. For example, virtually everyone is exposed to processed foods, but only some develop Type 2 diabetes, thus suggesting that they are carriers of specific polymorphisms that give rise to this disease. But, in fact, in the absence of processed foods, they would not be affected. For many years the consensus has been that metabolic syndrome, as well as several common cancers are caused by genetic factors, all based on the misinterpretation of this purely methodological paradox. There is no solution to this problem, but researchers and funding agencies need to be cognizant of this trap.

#### Level of detection (LOD) issue

Another important issue is related to the ability of our current epidemiological methods to provide a valid measurement of risk at low dose exposure, close to the “noise level” on the calibration curve. This issue has already been pointed out [10]. In the context of Toxicology, this methodological limitation remains largely ignored. Thus, inconsistent and incoherent results from studies on low dose exposures are not surprising.

#### Homogeneity versus external validity

Human environmental toxicology is mostly based on observational epidemiological studies, since randomized clinical trials with toxic agents is ethically unacceptable. Thus, most studies attempt to reduce the variability of known risk factors, which could interfere with the outcome. This “homogeneity” of study groups gives the chance of detecting tiny effects by reducing the “noise” from other factors. Frequently, environmental studies are done under very specific populations (i.e. immigrants, African-Americans, urban or rural groups). Some risk factors (i.e. poverty and associated lifestyle/nutritional patterns) are highly prevalent or almost ubiquitous in these study groups. By showing a correlation with a specific exposure, we have to wonder if we demonstrate the causal combination of environmental contaminants with an underlying lifestyle. Lifestyle, nutrition and epigenetic bagage are important determinants of health and biological resilience against environmental harms [11, 12]. Thus, results from a socially disadvantaged population cannot necessarily be translated to other populations. Different background characteristics influence the chance to observe statistically significant relationship and give an incorrect perception that epidemiological studies are non-reproducible. For example, the exposure to lead and other contaminants such as manganese or arsenic is higher in malnourished children because they absorb them more efficiently or eliminate less efficiently [13].

The variability in reported findings impedes risk assessment.

## Shifting from our current role of communicating “bad news” to a key role in developing bottom-up solutions

As environmental toxicologists, we recognize that our publications concerning the toxicity of environmental chemicals are communicated and sometimes sensationalized by the media and are thus generally perceived as “bad news” by the public. Unfortunately, citizens are almost never advised how to reduce exposure to “reasonable” levels. Simply creating panic or perhaps worse, skepticism, concerning environmental toxicants is a poor return on taxpayer dollars. The take-home message in most of our publications is that we need more research and we must to rely on government to manage the situation. When bureaucrats are interviewed by journalists, they adopt and cite the “lack of solid evidence”. There creates a vicious circle of nourishing the monster of evidence-based risk assessment, which requires more and more data.

## Conclusion: cutting out the middle man: the democratization of environmental toxicology

In contrast to classical Toxicology which has been dealing with toxic poisoning, we have to recognize that Environmental epidemiology is presently facing major methodological challenges which delay the production of valid knowledge on widespread low dose chemicals. As a result, studies are more costly, longer, and have low potential to be rapidly translated into an action, such as risk reduction.

By analogy with HIV epidemics, top-down strategies are more effective when the causal factor is well defined, geographically or demographically. However, once the causal factor has spread, only education and individual prevention works.

We cannot realistically expect that we will be able to provide a perfectly contaminant free environment when sources of these exposures are widely used in consumer products such as cosmetics/personal care products, food, packaging, and plastic or polymer containing goods. However, we have enough knowledge on sources of contaminants to help individuals make healthy choices. Where research can really help in the short term is in interventional trials on the best choices or lifestyle to adopt. By this we mean recruiting cohorts of families who accept to reduce their exposure to certain chemicals by restricting, for example, personal care products, and comparing them to matched controls. This is of particular importance for pregnant women and those planning pregnancies. These interventional trials would aim not only to reduce exposures but also to include nutritional interventions (e.g. one-carbon donors), which could help reduce damage to the developing embryo or fetus (e.g. folate supplementation to reduce arsenic exposure). Clinical interventional trials on simple, affordable solutions in families and individuals would also increase the credibility of our research among health care professionals, who will be able to translate this knowledge to the clinic.

The hard truth is that we live in a polluted world; we need to better understand biological neuroprotection against chemical toxicity, especially in developing fetuses and children. From this perspective, we have to prioritize a case-control approach in highly exposed subjects (i.e. study individuals who stay healthy despite high levels of contaminants and compare to those who perish). For neurodevelopmental disruptors, instead of, or in addition to, using clinical diagnoses (e.g. DSM-V) as outcomes, we suggest adopting the strategy proposed by the NIMH (National Institute of Mental Health), which recommends studying specific neurobehavioral phenotypes (Research Domain Criteria, RDoC). RDoC integrate several levels of information (from genomic, neuroimaging to behavior) to classify mental health in terms of specific biological dysfunctions [10].

We are working unceasingly like Sisyphus, pushing against environmental contaminants. But we are also spending tax dollars. Citizens want to know how to live in this polluted environment, and how to raise healthy children. We know that much of our exposure to chemicals is the result of our behavior as consumers. If citizens are ready to change their exposure and want affordable and feasible solutions – our duty is to test these solutions and use a community empowerment knowledge translation strategy.

We suggest that an independent research society, along the lines of the Environmental Working Group, should develop exposure guidelines and strategies to reduce exposure of vulnerable populations (e.g. pregnant women, children) to chemicals of concern such as BPA, flame retardants, phthalates, even before we have a complete description of their mechanism of action. They then need to provide this information directly to consumers and allow them to decide what action to take. Obviously, this strategy cannot substitute for the role of other stakeholders including regulatory agencies and public health scientists. No one wants to see an increase in NDD; however, bureaucracies tend to move slowly and require a high burden of proof before acting..

## Data Availability

not relevant.

## Abbreviations

ADHD: Attention Deficit/Hyperactivity Disorder
BPA: Bisphenol A
BPB: Bisphenol B
BPF: Bisphenol F
BPS: Bisphenol S
DSM-V: Diagnostic and statistical manual of mental disorders, 5th edition
NDD: Neurodevelopmental disorders
NIH: National institutes of health
NIMH: National institute of mental health
PBDEs: Polybrominated diphenyl ethers
RDoC: Research domain criteria

## Footnotes

1 Type III error: the null hypothesis is correctly rejected but for the wrong reason.
